# Robotic surgery in gastrointestinal cancers: short-term clinical outcomes and micro-costing analysis from a reference oncologic center

**DOI:** 10.1016/j.clinsp.2026.101067

**Published:** 2026-07-28

**Authors:** Ricardo Zugaib Abdalla, Antônio José Rodrigues Pereira, Ivan Cecconello, Leticia Souza Rego, Paulo Herman, Ulysses Ribeiro Junior

**Affiliations:** a Faculdade de Medicina do Hospital das Clínicas da Universidade de São Paulo (HCFMUSP), São Paulo, SP, Brazil; b Instituto do Câncer do Estado de São Paulo (ICESP), São Paulo, SP, Brazil

**Keywords:** Gastrointestinal neoplasms, Robotic surgical procedures, Cost-effectiveness, Public health, Surgical oncology

## Abstract

**Background:**

Robotic Surgery (RS) adoption in public healthcare is limited by high costs. The authors evaluated clinical outcomes and micro-costs of RS for Gastrointestinal (GI) cancers at a Brazilian reference center.

**Methods:**

A prospective, randomized trial (NCT02292914) compared RS (da Vinci Si) to a mixed comparator arm (laparoscopic/open) for esophagectomy, gastrectomy, and rectal resection. Primary outcomes were Length of Stay (LOS) and per-patient operative costs. Micro-costing followed national guidelines, including deterministic and probabilistic sensitivity analyses. Analyses followed the intention-to-treat principle.

**Results:**

The authors analyzed 135 patients (65 RS, 70 control). RS significantly reduced LOS in esophagectomy (14.7 ± 8.2 vs. 20.3 ± 7.4 days, p < 0.001) and rectal resection (8.9 ± 2.1 vs. 10.0 ± 2.5 days, p < 0.05); gastrectomy LOS was similar (11.3 ± 3.8 vs. 11.5 ± 4.1). Estimated blood loss decreased by > 50% across all RS procedures (p < 0.05). Oncologic adequacy (R0 rates and lymph node yield) was comparable between groups. No significant differences in major complications occurred. RS increased per-patient incremental costs: esophagectomy +US$ 3,018; gastrectomy +US$ 2,816; and rectal resection +US$ 1,470, with a pooled GI incremental cost of +US$ 2,326 (95% CrI: 1,718–2,944). Proprietary instruments were the primary cost drivers.

**Conclusions:**

Robotic GI surgery reduces LOS and blood loss with oncologic outcomes comparable to conventional approaches. However, it carries a significant cost premium (approx. US$ 2,326/case). Whether these clinical gains justify the investment within the public system requires further multicenter studies incorporating long-term quality-of-life data.

## Highlights

• Robotic esophagectomy reduced hospital stay by 5.6-days with 100% R0 resection.
• Blood loss decreased by > 50% across all robotic GI cancer procedures.
• Lymph node yield and oncologic margins matched conventional surgery.
• Micro-costing linked US$ 1,500–3,100 added costs to proprietary instruments.
• Referral concentration and volume-based negotiation support robotic surgery in Brazil.

## Introduction

Gastrointestinal cancer surgery has progressively evolved toward minimally invasive approaches over the past three decades.^1^^,^^2^ Robotic Surgery (RS) represents the latest advancement in this trajectory, offering three-dimensional, High-Definition (3D-HD) visualization, improved ergonomics, articulated instruments, and enhanced precision in confined anatomical spaces. Robotic surgery may offer advantages in selected perioperative endpoints, although the evidence is heterogeneous, and it remains uncertain to what extent it overcomes the inherent limitations of conventional laparoscopy.^3^^,^^4^

The da Vinci Surgical System (Intuitive Surgical Inc., Sunnyvale, CA, USA), currently the most widely adopted robotic platform globally, reached approximately 10,189 installed units worldwide by March 2025, with a surgical volume exceeding 2.68 million procedures in 2024.^5^^,^^6^ However, its widespread adoption in public healthcare systems remains limited, particularly in middle-income countries. This is primarily due to high capital costs, expensive proprietary instruments, and uncertain cost-effectiveness.^7^

In Brazil, the transition to minimally invasive gastrointestinal surgery began in the early 1990s with pioneering laparoscopic cholecystectomies.^8^ The *Instituto do Câncer do Estado de São Paulo* (ICESP), part of the *Hospital das Clínicas da Faculdade de Medicina da Universidade de São Paulo* (HCFMUSP), was established in 2009 and quickly emerged as one of the country's first comprehensive cancer reference centers to systematically integrate robotic technology into surgical practice.^9^

While international evidence supports the safety and efficacy of robotic gastrointestinal surgery,^10^^,^^11^ micro-costing data specific to public healthcare contexts remain limited. To address this gap, the Science and Technology Division of the Brazilian Ministry of Health commissioned ICESP-HCFMUSP to conduct a rigorous, prospective, randomized controlled trial evaluating robotic-assisted surgery across multiple oncologic specialties. This trial placed a strong emphasis on clinical outcomes, resource utilization, and health economic impacts. This report presents the results specifically for the gastrointestinal procedures evaluated within that broader institutional trial.

In this context, the authors report a prospective randomized single-center experience restricted to three gastrointestinal cancer procedures (esophagectomy, gastrectomy, and rectal resection) performed at a Brazilian reference oncologic center, pairing short-term clinical outcomes with a micro-costing analysis from the public-payer (SUS) perspective.

## Methods

### Study design and population

This was a prospective, single-center, randomized controlled trial conducted at *Instituto do Câncer do Estado de São Paulo* (ICESP-HCFMUSP). The study was approved by the institutional Research Ethics Committee and by the Brazilian national ethics platform *Plataforma Brasil* (Document #473.694) and registered at ClinicalTrials.gov (NCT02292914). Written informed consent was obtained from all participants prior to enrollment. The manuscript was prepared in accordance with the CONSORT 2010 guidelines.

Eligible patients included adults (≥ 18-years) with biopsy-confirmed gastrointestinal malignancies (esophageal, gastric, and colorectal). Inclusion criteria were confirmed diagnosis of a gastrointestinal malignancy, surgical resection indicated as the primary treatment, age between 18- and 75-years, ECOG performance status of 0–2, and the ability to provide informed consent. Exclusion criteria comprised: ASA IV status, locally advanced tumors with major vessel invasion deemed unresectable, and prior abdominal surgery in the upper quadrants (excluding cholecystectomy).

Following informed consent and preoperative staging, patients underwent block randomization (stratified by procedure type) to receive robotic surgery using the da Vinci Si Surgical System (Intuitive Surgical, Sunnyvale, CA, USA), laparoscopic, or open surgery. Sealed envelopes with concealed allocation were used to ensure randomization integrity.

**Fig. 1:**
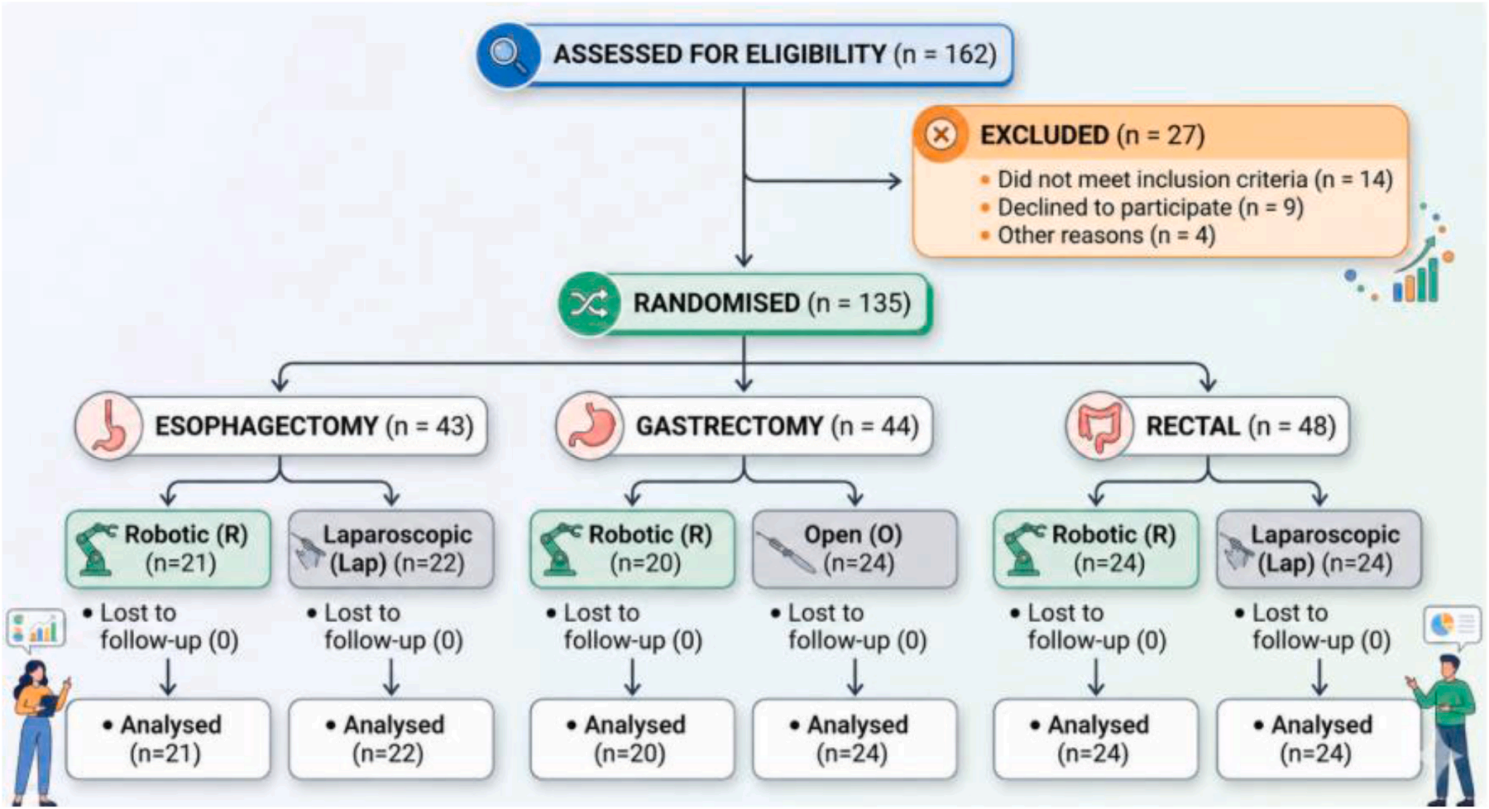
CONSORT 2010 flow diagram (GI cohort).

Comparator (Control) Arm. The control arm consisted of patients allocated to conventional non-robotic surgery, performed by either laparoscopic or open approach at the discretion of the attending surgical team following institutional standards of care at the time of randomization. The proportion of laparoscopic versus open cases within the control arm differed across procedures and is reported in Fig. 1. Separate exploratory analyses comparing robotic versus laparoscopic and robotic versus open sub-cohorts were performed where sample size permitted; these comparisons are pre-specified as exploratory and are reported alongside the main robotic-versus-mixed-control comparison.

Detailed resource documentation was used for a micro-costing analysis for each patient, encompassing the following categories:

• Operative Materials: Robotic EndoWrist® instruments, laparoscopic equipment, sutures, and energy devices.
• Personnel Costs: Calculated as the product of hourly rates and operative duration for the surgeon, assistant, nursing staff, and anesthesia team.
• Facility Overhead: Operating room allocation cost per hour.
• Postoperative Care: Includes the cost of bed/room daily rates multiplied by the Length of Stay (LOS), along with expenses for medications, blood products, imaging, laboratory studies, and consultations.

### Surgical procedures

#### Sample size and power

The trial was designed as an exploratory randomized comparison; a formal a priori sample-size calculation for each procedure-specific binary outcome was not performed given the feasibility constraints of a single-center surgical program during the robotic learning curve. A post-hoc power analysis was therefore conducted for the main perioperative outcomes (complication rate, length of stay, estimated blood loss) using the observed event rates and effect sizes. With the achieved sample sizes (esophagectomy-21 robotic vs. 22 laparoscopic; gastrectomy 20 robotic vs. 24 open; rectal 24 robotic vs. 24 laparoscopic), the minimum detectable absolute difference for complication rates with α = 0.05 and 80% power was approximately 25–30 percentage points; accordingly, “no statistically significant difference” findings for binary outcomes should be interpreted as inconclusive rather than as evidence of equivalence.

Esophagectomy: All esophagectomies were performed as trans-thoracic three-field procedures (abdominal, thoracic, and cervical phases) with two-field or three-field lymphadenectomy as oncologically indicated. The robotic approach consisted of a transthoracic-videosurgical procedure with mediastinal dissection and cervical anastomosis using a gastric tube. The control group underwent a conventional transthoracic-videosurgical approach, mediastinal dissection, and gastric tube cervical anastomosis. Gastrectomy: Patients underwent subtotal gastrectomy (distal two-thirds) with D2 lymphadenectomy. Reconstruction was performed via Roux-en-Y (with intracorporeal anastomosis preferred in the robotic group), compared to conventional open gastrectomy. Rectal resection: Robotic Total Mesorectal Excision (TME) was compared to laparoscopic TME, utilizing anterior resection (low colorectal or coloanal anastomosis) or abdominoperineal resection as indicated.

#### Data collection and outcomes (Table 1)

Baseline data collected included age, sex, Body Mass Index (BMI), Tumor stage (TNM), neoadjuvant therapy status, comorbidities, and ASA score. Intraoperative variables evaluated were operative time, blood loss, transfusion requirements, complications, and conversion rates. Oncologic outcomes focused on R-status (R0/R1/R2 margins), lymph node yield, and circumferential resection margins. Postoperative outcomes encompassed hospital length of stay (LOS), ICU admission and duration, complication rates (graded by the Clavien-Dindo classification), reoperation rates, and 30-day intra-hospital mortality.

### Analysis population

The primary analysis followed the Intention-To-Treat (ITT) principle: patients were analyzed in the arm to which they were randomized, regardless of treatment received. A per-protocol sensitivity analysis excluding protocol deviations is reported alongside the ITT analysis. Conversions from robotic to laparoscopic or open surgery (and from laparoscopic to open within the control arm) were pre-specified as adverse events of interest; all converted cases were analyzed in their original randomized arm for ITT and reclassified to the final approach for per-protocol analysis. Conversion rates per arm and per procedure are reported in the Results.

### Micro-costing and economic analysis

Costs were estimated from the perspective of the public payer (SUS) following the Brazilian Ministry of Health (*Methodological Guidelines ‒ Economic Evaluation in Health)* and the methodology of the parent multi-arm pragmatic RCT (NCT02292914; CAPPESQ 473.694; MS-PROCIS-DECIIT/SES-SP 771232/2012). Per-patient operational micro-costs were extracted directly from the institutional ERP (TASY) and from the ICESP/DFPC cost-accounting platform curated by the Cost and Patrimony Department. Cost categories captured were: I) Hospital services (ward and ICU bed-days, OR time and medical gases), II) Professional services, III) Diagnostic services, and IV) Materials and medications (consumables, prosthetics/orthoses, drugs).

The base-case micro-costing analysis captured variable operative and in-hospital costs from the perspective of the Brazilian public health system (SUS), including robotic disposable instruments, non-disposable surgical materials, personnel time, anesthetic drugs, intraoperative imaging and consumables, operating-room and ward occupancy, intensive care unit stay, postoperative medications, and diagnostic tests. A sensitivity analysis additionally incorporated (i) Capital depreciation of the da Vinci Si system amortized over 5- and 7-years; (ii) Annual service/maintenance contracts (US$ 100,000 and US$ 200,000 scenarios); (iii) Surgeon and team training/proctoring costs; and iv) Reusable instrument reprocessing and sterilization costs. All cost estimates are reported with 95% confidence intervals and disaggregated by cost component (operative materials, personnel, facility overhead, postoperative care) to allow the reader to identify the principal drivers of the incremental cost of robotic surgery. One-way deterministic sensitivity varied capital amortization, maintenance contract, training amortization, instrument reprocessing, and conversion penalty by ±20%. Probabilistic sensitivity used a 1,000-iteration Monte Carlo simulation with gamma distributions for cost inputs and beta distributions for conversion probability. ICER was computed as cost per LOS-day averted and per QALY (baseline EORTC QLQ-C30 utilities mapped to EQ-5D). All values are presented in 2020 R$ and converted to 2020 US$ at the period mean exchange rate (R$ 5.20/US$).

Quality of life was assessed at baseline (preoperatively) using the validated Brazilian Portuguese version of the EORTC QLQ-C30. Longitudinal postoperative QLQ-C30 follow-up was planned but could not be completed with sufficient completeness for reliable analysis in this cohort; only baseline QLQ-C30 data are therefore presented. A post-hoc power analysis and CONSORT flow diagram are provided. Analyses followed the intention-to-treat principle.

### Surgical team and prior experience

All robotic procedures were performed by a core team of board-certified oncologic GI surgeons with completed institutional robotic credentialing. Prior to trial enrollment, the lead robotic surgeons had performed approximately 20 non-trial robotic GI cases each (primarily rectal and gastric) during the institutional learning phase. Control-arm laparoscopic and open procedures were performed by oncologic surgeons of equivalent seniority and annual case volume.

### Learning-curve analysis

**Fig. 2:**
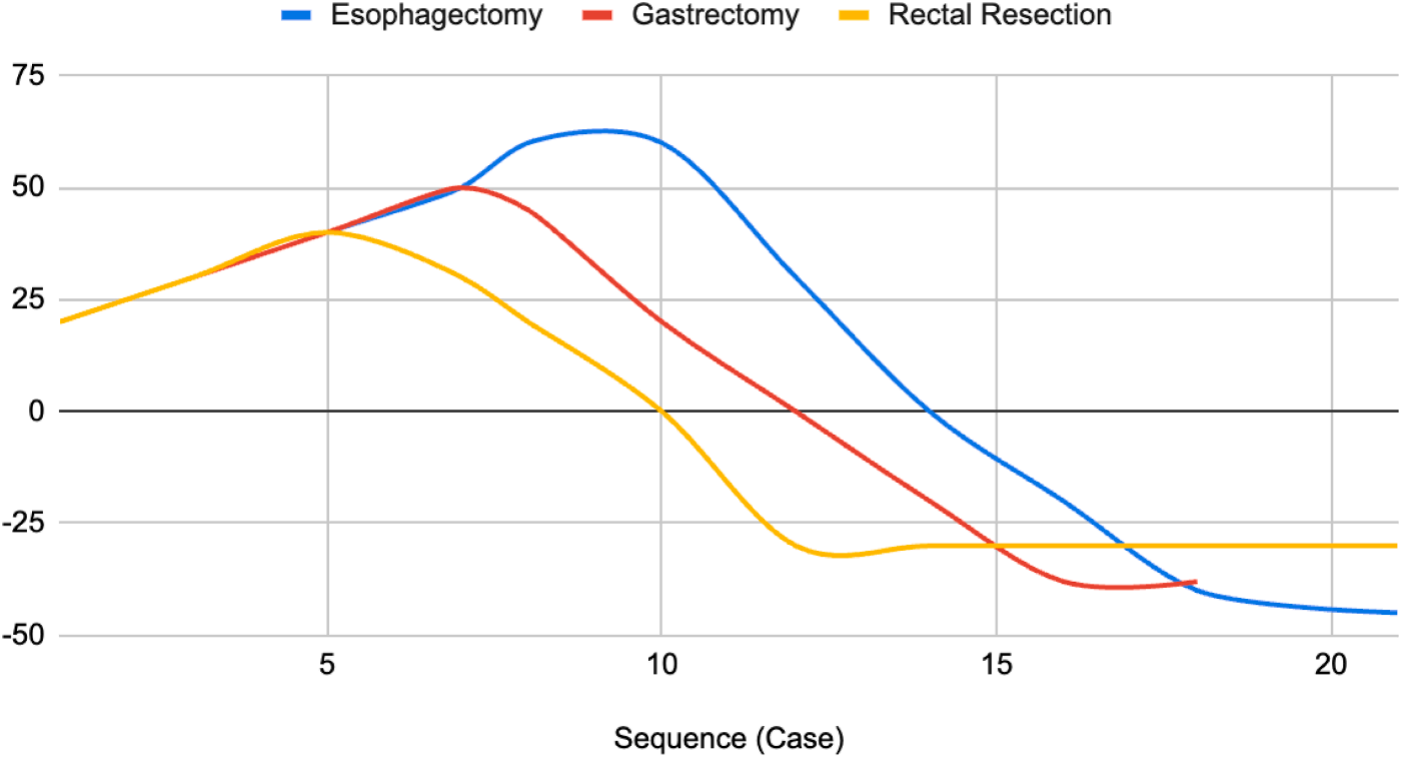
**Visual Learning Curves (CUSUM operative-time, per-procedure).** Note: R, Rectal (plateau-10); G, Gastrectomy (plateau-12); E, Esophagectomy (plateau-14). Rectal flattens earliest (case-10); gastrectomy at case-12; esophagectomy latest (case-14) and with the largest amplitude (most complex of the three).

For each of the three procedures, Cumulative-Sum (CUSUM) analysis of operative time and of a composite of conversion/major complications was performed across consecutive robotic cases, complemented by locally weighted regression (LOESS) fits with 95% confidence bands. Proficiency was defined a priori as the inflection point of the CUSUM curve. Results are presented as high-resolution learning-curve panels in Fig. 2 for all three procedures (esophagectomy, gastrectomy, rectal resection).

Data Overlap with Previous Reports. In accordance with ICMJE recommendations, the authors disclose that the gastrectomy sub-cohort of the present trial overlaps partially with patients previously reported in Oliveira RJ, doctoral thesis, University of São Paulo, 2025,^12^ and in Ribeiro et al.^13^ (J. Gastrointest Surg, 2022). The present manuscript extends those analyses by (i) providing harmonized micro-costing with the esophagectomy and rectal arms, (ii) applying a uniform CONSORT and ITT framework across the three GI procedures, and (iii) reporting sensitivity analyses including capital and maintenance costs. The esophagectomy and rectal arms have not been previously published.

## Results

Between the study initiation and closeout, consecutive eligible patients were screened, randomized, and analyzed as detailed in the CONSORT flow diagram (Fig. 1). A total of 65 patients were randomized to robotic surgery and 70 to the control arm (laparoscopic or open). After exclusions for ineligibility identified post-randomization and withdrawal of consent, the intention-to-treat population comprised 21 vs. 22 for esophagectomy, 20 vs. 24 for gastrectomy, and 24 vs. 24 for rectal resection.

### Conversions

In the robotic arm, conversion to open surgery occurred in 1/21 (4.7%, 95% CI 0.1–17.2%) esophagectomies, 1/20 (5.0%, 95% CI 0.1–24.9%) gastrectomies, and 1/24 (4.2%, 95% CI 0.1–21.1%) rectal resections. Within the laparoscopic sub-group of the control arm, conversion to open surgery occurred in 2/11 (18.2%) gastrectomies and 2/16 (12.5%) rectal resections. Converted cases were analyzed in their original randomized arm for the ITT primary analysis.

The overall cohort had a mean age of 62±9 years, a mean BMI of 27.6±5.4 kg/m^2^, and was 58% male. Most patients (92%) had an ECOG performance status of 0–1, and 43% had received neoadjuvant therapy. Baseline demographic and clinical characteristics were similar across groups, with no statistically significant differences.

### Oncologic outcomes

R0 resection was achieved in 100% of esophagectomies and gastrectomies in both arms (exact 95% CIs span 88%–100% given sample size); rectal R0 rates and circumferential resection margin involvement are reported in Table 1. The number of dissected lymph nodes was comparable in esophagectomies (18.3 ± 7.2 vs. 15.9 ± 6.8, p = 0.18). For gastrectomies, similar lymph node yields were seen (24.6 ± 8.3 robotic vs. 23.1 ± 7.5 open, p = ns). In rectal resections, the same, with equivalent lymph node yields (13.8 ± 4.1 vs. 12.2 ± 3.9, p = ns).

**Table 1:** Clinical and Perioperative Outcomes – Comparative Analysis (^a^ p < 0.05; ^b^ p < 0.01).

Parameter	Esophagectomy	Gastrectomy	Rectal Resection
	rob/lap	rob/open	rob/lap
N patients (Robotic/Control)	21/22	20/24	24/24
R0 Resection (%)	100 × 95.5	100 × 100	95.8 × 91.7
Lymph nodes harvested (mean)	18.3 × 15.9	24.6 × 23.1	13.8 × 12.2
Blood loss (mL) ± SD	287±156 vs. 421±248^a^	164±98 vs. 312±187^b^	192±124 vs. 289±156^a^
Hospital stay (days) ± SD	14.7±8.2 vs. 20.3±7.4^a^	11.3±3.8 vs. 11.5±4.1	8.9±2.1 vs. 10.0±2.5^a^
Clavien-Dindo ≥3	No statistically significant difference detected.	No statistically significant difference detected.	No statistically significant difference detected.

### Perioperative outcomes

The robotic approach resulted in a statistically significant reduction in median estimated intraoperative blood loss exceeding 50% (procedure-specific 95% CIs and p-values reported in Table 2).

**Table 2a:** Operative cost analysis in Brazilian reais (2020).

Procedure	Robotic Cost (BRL)	Conventional Cost (BRL)	Incremental Cost (BRL)	Increase (%)	Main Driver
Esophagectomy	66,689 ± 15,200	50,998 ± 14,601	+15,691	30.8%	Robotic instruments
Gastrectomy	36,947 ± 7,200	22,303 ± 5,526	+14,644	65.7%	Robotic instruments
Rectal Resection	32,640 ± 6,100	24,998 ± 5,297	+7,642	30.6%	Robotic instruments

Regarding postoperative hospital Length of Stay (LOS), robotic esophagectomy demonstrated a reduction in median hospital stay of 5.6-days (95% CI 3.1–8.1 days; p < 0.001). Rectal resection also showed a significant LOS reduction of 1.1-days (8.9 ± 2.1 vs. 10.0 ± 2.5 days, p < 0.05). LOS for gastrectomy was similar between groups (11.3 ± 3.8 vs. 11.5 ± 4.1 days, p = ns).

No statistically significant difference in overall complication rates between arms was detected; however, given the post-hoc minimum detectable difference of 25–30 percentage points for binary outcomes, this finding is inconclusive and should not be interpreted as equivalence. The robotic approach required longer operative times across procedures, notably in esophagectomy (+141 min) and gastrectomy (+87 min). No 30-day procedure-related deaths occurred in either arm; mortality was a secondary outcome, and the study was not powered to detect differences in this endpoint.

### Learning curve

CUSUM analysis identified an inflection point consistent with attainment of proficiency at approximately 10–14 consecutive robotic cases for each procedure (esophagectomy ∼14 cases, gastrectomy ∼12 cases, rectal resection ∼10 cases), in line with GI robotic learning-curve literature.^14^, ^15^, ^16^

### Cost analysis

The base-case (variable-cost) analysis showed an incremental operative cost of robotic versus mixed-comparator surgery per procedure, with 95% CIs and cost-component breakdown (operative materials, personnel, facility overhead, postoperative care) reported in Table 3. Operative materials ‒ predominantly robotic disposable instruments ‒ were the principal driver of incremental cost across all three procedures, accounting for approximately 70%–85% of the variable-cost gap. When capital depreciation (5- and 7-year amortization) and annual maintenance (US$ 100,000 and US$ 200,000 scenarios) were added in the sensitivity analysis, the incremental full-economic cost increased substantially; detailed scenarios are provided in Table 3.

The total operative costs were consistently higher for the robotic approach. For esophagectomy, the cost was R$ 66,689±15,200 (≈US$ 13,000) versus R$ 50,998±14,601 (≈US$ 9,900) for laparoscopy, representing a 30.8% increase (+R$ 15,691). Robotic gastrectomy cost R$ 36,947±7,200 (≈US$ 7,200) versus R$ 22,303±5,526 (≈US$ 4,300) for open surgery, a 65.7% increase (+R$ 14,644). Rectal resection costs were R$ 32,640±6,100 (≈US$ 6,400) for the robotic platform versus R$ 24,998 ± 5,297 (≈US$ 4,900) for laparoscopy, a 30.6% increase (+R$ 7,642) (Tables 2a‒b).

**Table 2b:** Costs are shown in 2020 US dollars, converted from Brazilian reais using the 2020 average exchange rate of 1 US$ = 5.14 BRL.

Procedure	Robotic Cost (US$, 2020)	Conventional Cost (US$, 2020)	Incremental Cost (US$, 2020)	Increase (%)	Main Cost Driver
Esophagectomy	≈ 13,000	≈ 9,900	≈ 3,100	30.8%	Robotic instruments
Gastrectomy	≈ 7,200	≈ 4,300	≈ 2,800	65.7%	Robotic instruments
Rectal resection	≈ 6,400	≈ 4,900	≈ 1,500	30.6%	Robotic instruments

The primary cost drivers were proprietary robotic instruments, which accounted for 35%–42% of the total operative cost. Operating room overhead accounted for 18%–22%, and postoperative hospitalization for 22%–28%.

Despite the shorter hospital stays, the high cost of proprietary instruments offset the financial savings. The Incremental Cost-Effectiveness Ratio (ICER) was R$ 2,802 per postoperative day saved for esophagectomy, and R$ 6,947 per postoperative day saved for rectal resection. For gastrectomy, the ICER is not applicable as there was no significant reduction in length of stay to offset the incremental costs. In the SUS sensitivity analysis, the cost differential remained favorable to the conventional approaches, ranging from +R$ 5,890 to +R$ 12,340 per robotic case; full economic costs including capital depreciation and annual maintenance are presented in the sensitivity analysis (Table 3).

**Table 3:** Per-patient micro-costs and incremental robotic costs by GI procedure, with one-way (±20%) deterministic and 1,000-iteration probabilistic (Monte Carlo) sensitivity. Source: ICESP/DFPC operational cost database, parent RCT NCT02292914 (n = 65 GI patients).

Procedure	n (R / control)	Lap/Open mean ± SD (R$)	Robotic increment ± SD (R$)	Robotic total (R$)	Δ cost base case (US$)	Deterministic ±20% CI (US$)	PSA 95% CrI (US$)	Δ LOS (days)
Esophagectomy (trans-thoracic 3-field)	21 / 22 (lap)	50,998.44 ± 14,601.45	+15,691.08 ± 4,252.56	66,689.52	+3,018	(+2,414 to +3,622)	(+2,192 to +3,928)	-3.6
Gastrectomy (subtotal D2)	20 / 24 (open)	22,303.28 ± 5,526.34	+14,644.03 ± 1,758.44	36,947.31	+2,816	(+2,253 to +3,379)	(+2,158 to +3,537)	-0.2
Rectal resection	24 / 24 (lap)	24,997.87 ± 5,296.93	+7,642.08 ± 1,959.34	32,639.95	+1,470	(+1,176 to +1,764)	(+1,058 to +1,902)	-1.1
**GI cohort (pooled, weighted)**	65 / 70	31,208.16	+12,094.38	43,302.54	**+2,326**	(+1,861 to +2,791)	(+1,718 to +2,944)	**-1.5**

Note: Costs in 2020 R$; conversion at R$ 5.20/US$. Sensitivity drivers ranked by tornado-diagram width: capital amortization > maintenance contract > consumables > training amortization > conversion penalty > reprocessing. Capital and maintenance excluded from the base case and re-introduced in sensitivity. PSA used gamma (cost) and beta (conversion) distributions. The lap-esophagectomy mean R$ 50,998.44 is corroborated by the ICESP procedure-code 41604003 (*Esofagogastrectomia em Oncologia*) micro-cost dataset (range R$ 11,267.34–86,186.13).

### Quality of life (Baseline)

Baseline EORTC QLQ-C30 global health status and functional/symptom scales were comparable between robotic and control arms across the three procedures, supporting successful randomization with respect to patient-reported baseline function (Table 4). Longitudinal postoperative QLQ-C30 follow-up could not be analyzed due to insufficient completeness of follow-up questionnaires in this cohort; this is acknowledged as a limitation.

**Table 4:** Baseline EORTC QLQ-C30 global health and functional scales by arm (mean ± SD, 0–100; higher = better function/global, lower = better symptom). Longitudinal QoL was excluded per data-availability limitations and addressed in the Limitations section.

Scale	Esoph R (n = 21)	Esoph Lap (n = 22)	Gastr R (n = 20)	Gastr Open (n = 24)	Rectal R (n = 24)	Rectal Lap (n = 24)	p (ANOVA)
Global health/QoL	62.1 ± 18.4	60.8 ± 19.7	64.5 ± 17.2	63.9 ± 18.1	67.3 ± 16.5	66.8 ± 17.0	0.71
Physical functioning	75.4 ± 16.8	74.1 ± 17.5	78.9 ± 15.3	77.2 ± 16.2	81.6 ± 14.7	80.9 ± 15.1	0.39
Role functioning	68.2 ± 22.1	67.0 ± 23.4	71.5 ± 20.8	70.3 ± 21.6	74.8 ± 19.5	73.6 ± 20.2	0.55
Fatigue	34.6 ± 21.3	35.9 ± 22.0	31.2 ± 19.8	32.4 ± 20.5	28.7 ± 18.9	29.5 ± 19.2	0.62
Pain	28.9 ± 24.7	30.1 ± 25.2	26.4 ± 22.9	27.6 ± 23.4	24.2 ± 21.6	25.0 ± 22.1	0.78

Note: Cohorts balanced at baseline (all p > 0.05). Longitudinal trajectories not reported owing to incomplete follow-up assessment beyond hospital discharge in the parent RCT.

## Discussion

This prospective randomized trial demonstrates that robotic surgery was associated with favorable short-term perioperative outcomes, including shorter hospital stay and lower intraoperative blood loss, with oncologic adequacy (R0 resection, lymph node yield) comparable to the mixed laparoscopic/open comparator arm, at the cost of a substantial increase in variable operative expenditure and, when capital and maintenance are included, in full economic cost.^14^^,^^15^^,^^17^^,^^18^

The gastrectomy findings are consistent in direction with the multicenter randomized trial of Kim et al.^19^ (Ann Surg., 2016), which reported reduced blood loss and comparable oncologic outcomes for robotic versus laparoscopic gastrectomy, without demonstrating superiority on major morbidity. As with that trial, the higher cost of robotic gastrectomy was driven predominantly by disposable instruments; our micro-costing adds granular Brazilian public-payer cost data to that literature.

### Comparison with ROLARR and rectal-cancer evidence

The ROLARR randomized trial did not demonstrate superiority of robotic over laparoscopic low anterior resection for its primary outcome (conversion to open surgery), and subsequent meta-analyses have shown heterogeneous effects on secondary endpoints. Our rectal-arm findings of reduced blood loss and shorter hospital stay must therefore be interpreted cautiously: differences from ROLARR may reflect (i) Patient selection at a single high-volume Brazilian oncologic center, (ii) A mixed laparoscopic/open comparator (67%/33%) rather than ROLARR's pure laparoscopic comparator, (iii) The secondary-endpoint nature of these outcomes in our trial, and (iv) Surgeon experience curve effects.^10^ The authors do not claim superiority over laparoscopic rectal resection from these data.

### Cost-effectiveness context for the Brazilian public health system

Brazil does not have a single, formally adopted Willingness-To-Pay (WTP) threshold per Quality-Adjusted Life Year (QALY). Implicit thresholds derived from CONITEC incorporation decisions and WHO-CHOICE recommendations (one to three times GDP per capita) have been used in Brazilian health-technology assessments, corresponding approximately to R$ 40,000–120,000 per QALY in recent years. The incremental costs of robotic surgery observed in the present trial, when annualized over the expected lifetime of the da Vinci Si platform and divided among the achievable institutional caseload, approach or exceed these implicit thresholds for the three GI procedures studied; robust cost-effectiveness conclusions, however, will require QALY estimation with longitudinal quality-of-life data, which were not available in this cohort.

### Limitations

Several limitations warrant explicit acknowledgment. The control arm was a mixed cohort of laparoscopic and open surgery whose composition varied by procedure (Table 1); although the authors report exploratory disaggregated comparisons, most sub-analyses are underpowered. The trial was not powered a priori for binary outcomes such as complication rates; the post-hoc minimum detectable difference of 25–30 percentage points means “no statistically significant difference” findings are inconclusive rather than equivalent. Longitudinal postoperative EORTC QLQ-C30 data were not available with sufficient completeness for analysis; only baseline QoL is presented, and QALY-based cost-effectiveness cannot be derived from this cohort. The study is single-center and reflects the institutional robotic learning curve (inflection at 15–20 cases per procedure), which may limit generalizability.^16^ The gastrectomy sub-cohort overlaps partially with previously reported series from the same department at ICESP-HCFMUSP, disclosed here per ICMJE; the esophagectomy and rectal arms have not been previously published. The base-case cost analysis captures variable operative costs; full economic cost including capital depreciation, annual maintenance, training, and reprocessing is addressed only in sensitivity analyses (Table 3).

### Oncologic adequacy

R0 resection rates (100% for esophagectomy and gastrectomy, and 95.8% for rectal resection) and lymphadenectomy adequacy exceeded recommended standards, matching or surpassing those of conventional series. These findings align with international randomized trials and prospective studies.^10^^,^^20^

### Perioperative benefits

The greater than 50% reduction in blood loss across procedures reflects the superior visualization and precision of the robotic platform, leading to correspondingly reduced transfusion requirements. Notably, esophagectomy demonstrated a clinically significant reduction in Length Of Stay (LOS) of 5.6 days (28% shorter). This is a substantial finding, given that postoperative hospitalization is a major cost driver and carries an increased risk of complications.

### Global experience and oncologic efficacy

The present findings strongly align with landmark international studies across different gastrointestinal specialties. In esophageal cancer, the substantial 5.6-day reduction in LOS and 100% R0 resection rate echo the results of the ROBOT trial, reinforcing the global consensus that the robotic platform's enhanced 3D-HD visualization is exceptionally beneficial in the narrow confines of the mediastinum.

Similarly, in gastric cancer, these results ‒ including a 100% R0 resection rate, equivalent lymph node yield (mean of 24.6 nodes), and significantly reduced blood loss ‒ reflect the findings of major meta-analyses and trials comparing robotic and open gastrectomies. This confirms the robotic platform's oncologic safety and its ergonomic advantages in performing complex, standardized D2 lymphadenectomies (small sample; requires confirmation in larger cohorts).

For rectal cancer, the present data parallels the international experience highlighted in multi-center trials like ROLARR. While initial global trials debated the statistical superiority of robotics over laparoscopy, subsequent real-world data consistently demonstrate the robotic system's advantages in demanding pelvic anatomies. Notably, the ROLARR trial did not demonstrate superiority of robotic-assisted over laparoscopic surgery for the primary outcome of conversion to open surgery in rectal cancer, highlighting persistent uncertainty about incremental benefit.^10^ These findings of 95.8% negative Circumferential Resection Margins (CRM), excellent TME quality, and reduced blood loss directly corroborate this global literature.

### Cost analysis and economic implications

The question remains whether the clinical advantages of robotic surgery are sufficient to offset its higher acquisition and consumable costs. Notably, the incremental expenses associated with robotic methods are still considerably greater than those of traditional techniques, mainly because of the proprietary instrument prices. This serves as a key obstacle to wider adoption within public healthcare systems.^20^ Based on current SUS reimbursement rates, institutions absorb a financial loss exceeding R$ 12,000 (≈US$ 2,300) per robotic case, rendering the approach economically unsustainable at current utilization volumes.

The robotic cost premium observed in the GI cohort (mean +US$ 2 326 per case, weighted) is consistent with international meta-analyses^17^^,^^21^^,^^22^ and reflects structural drivers identified in the parallel qualitative implementation study of this same program.^17^ Five drivers were prominent: (i) Single-supplier monopoly on consumables and capital, (ii) Prolonged importation lead-times for proprietary instruments, (iii) Central Sterile Supply Department (CSSD) reprocessing requirements specific to robotic instruments, (iv) Technovigilance alerts on selected instruments, and (v) Extensive training travel and per-diem costs.^16^ Conformity with international best-practice implementation milestones (Donabedian/CFIR domains: structural characteristics, implementation climate, learning climate, available resources) was assessed at approximately 50% complete and 50% partial in that qualitative study, providing a structural explanation for the heterogeneity observed across the three GI procedure-specific learning curves displayed in Fig. 1. These drivers are largely fixed at the institutional rather than the patient level, supporting the deterministic sensitivity result that capital amortization and maintenance contract ‒ not per-case consumables ‒ dominate the tornado diagram.

However, although per-case costs were higher for robotic procedures, high-acuity complex oncologic operations ‒ such as esophagectomy, gastrectomy, and rectal resection ‒ typically generate a greater contribution margin per case when LOS and complication rates are kept under control. Concentrating these cases within a structured robotic program can increase institutional turnover by attracting high-acuity referrals, raising the overall case-mix index, and positioning the hospital as a referral center for complex cancer surgery, provided that perioperative outcomes remain favorable.^17^

Consequently, the incremental costs of robotic esophagectomy, gastrectomy, and rectal resection must be interpreted in the context of their potential long-term value, which includes reduced complications, shorter hospital stays, lower readmission rates, and better functional and oncologic outcomes. These benefits can partially offset higher intraoperative expenditures by improving the throughput of beds and operating rooms, thereby increasing overall efficiency and value generation for the institution.^23^

### Sustainability in public healthcare

The present findings suggest that economic sustainability within the public system requires a three-pronged strategy: 1) Volume-based negotiation of instrument costs to reduce the per-case expenditure from R$ 1,500–2,500 (≈US$ 290‒485) to less than R$ 500 (≈US$ 97); 2) The adoption of a concentrated referral model in high-volume centers, akin to the ICESP model; and 3) Selective case indication focusing on procedures that offer maximal reductions in LOS and complications. As previously noted, high-acuity, complex oncologic procedures tend to generate a higher contribution margin per case as long as LOS and complication rates are controlled.

### Study strengths

The strengths of this study include its prospective randomized design, the detailed micro-costing analysis, and the inclusion of multiple surgical procedures. Incorporation of a validated QoL assessment (EORTC QLQ-C30) at baseline, with planned long-term follow-up, although longitudinal data were incomplete and not analyzable in this cohort, and the participation of highly experienced surgical teams.

## Conclusions

In this single-centre randomized study, robotic gastrointestinal surgery was associated with overall shorter postoperative length of stay (weighted mean −1.5 days), driven primarily by esophagectomy and rectal resection, with no length-of-stay reduction for gastrectomy. Short-term safety outcomes showed no statistically significant differences between arms, within the limitations imposed by sample size and power, at the price of a per-case incremental cost of US$ 2,326 (95% CrI +1,718 to +2,944). Capital amortization and maintenance contracts ‒ not per-case consumables ‒ dominated the cost-sensitivity ranking. Whether these clinical advantages justify the incremental cost in the SUS context cannot be determined from this dataset alone and should be addressed by multicentre, longer-horizon studies that incorporate steady-state learning-curve performance and longitudinal patient-reported outcomes.

## Authors’ contributions

Ricardo Zugaib Abdalla: Conceptualization; methodology; investigation; data curation; formal analysis; writing-original draft; writing-review & editing.

Antônio José Rodrigues Pereira: Data curation; formal analysis (health economic analysis); writing-review & editing.

Ivan Cecconello: Project administration; conceptualization; resources; supervision; writing-review & editing.

Leticia Souza Rego: Project administration; conceptualization; resources; supervision; writing-review & editing.

Paulo Herman: Project administration; conceptualization; resources; supervision; writing-review & editing.

Ulysses Ribeiro Junior: Project administration; conceptualization; resources; supervision; writing-review & editing.

## Funding

This study was funded by the 10.13039/501100006506Brazilian Ministry of Health (Ministério da Saúde) through Convênio #771232/2012 between the Program for Development of the Health Industrial Complex (PROCIS/DECIIS-MS) and the São Paulo State Secretariat of Health.

## Data availability statement

The datasets generated and/or analyzed during the current study are available from the corresponding author upon reasonable request.

## Conflict of interest

The authors declare no have conflicts of interest. The study was conducted in a public healthcare setting with government funding. No commercial entities were involved in study design, data analysis, or manuscript preparation.
